# Salvianolic Acid A Mitigates Osteoporotic Bone Loss by Repressing Reactive Oxygen Species via the Nrf2–HO‐1 Pathway

**DOI:** 10.1002/ptr.8503

**Published:** 2025-08-06

**Authors:** Hao Qiu, Chenhui Cai, Ying Zhang, Sizhen Yang, Xu Hu, Tongwei Chu

**Affiliations:** ^1^ Department of Orthopedics Xinqiao Hospital, Army Medical University Chongqing China

**Keywords:** NFATc1, NRF2, Osteoclast, ROS, SAA

## Abstract

Osteoporosis, characterized by osteoclastic bone resorption, has been the focus of research. Studies implicate that reactive oxygen species (ROS) accumulate intracellularly during osteoclastogenesis. Salvianolic acid A (SAA), a compound derived from *Salvia miltiorrhiza*, has been widely used to treat cardiovascular and cerebrovascular disorders, owing to its antioxidant and anti‐inflammatory properties. In this study, we investigated the therapeutic effects of SAA on osteoporotic bone loss in vitro and in osteoporotic mice induced by ovariectomy (OVX) and explored the underpinning mechanisms. In vitro, SAA significantly restrained osteoclastogenesis and osteoclastic resorption in a dose‐ and time‐dependent manner. SAA markedly blocked the expression of osteoclast‐specific genes and proteins such as NFATc1 and c‐Fos. Specifically, SAA reduced ROS production by enhancing the expression and nuclear translocation of nuclear factor erythroid 2‐related factor 2 (Nrf2) to activate HO‐1 and catalase, with no effect on Bach1. In addition, SAA simultaneously suppressed the NF‐κB and mitogen‐activated protein kinase (MAPK) signaling pathway, ultimately arresting NFATc1 expression to constrain the differentiation and function of osteoclasts. Micro‐CT and histological evidence demonstrated that SAA at a nontoxic dose successfully reduced bone loss induced by OVX, with fewer mature osteoclasts. These findings revealed that SAA provides a potential treatment strategy for reducing osteoclast‐related bone ailments, including osteoporosis.

**Abbreviations**
Atp6v0d2: ATPase H^+^ transporting V0 subunit D2
BMMs: bone marrow macrophages
BS: bone surface
BV/TV: bone volume per tissue volume
c‐Fos: proto‐oncogene c‐Fos
Ctsk: cathepsin K
H&E: hematoxylin and eosin
MAPK: mitogen‐activated protein kinase
M‐CSF: macrophage colony stimulating factor
Mmp9: matrix metallopeptidase 9
N.Oc: number of osteoclasts
NFATc1: nuclear factor of activated T cells 1
NF‐κB: nuclear factor kappa light chain enhancer of activated B cells
Nrf2: nuclear factor erythroid 2‐related factor 2
NS: nonsignificant
Oc.S: osteoclast surface
RANKL: receptor activator of nuclear factor‐κB ligand
ROS: reactive oxygen species
SAA: salvianolic acid A
TB: trabecular bone
Tb.N: trabecular number
Tb.Th: trabecular thickness
TRAcP: tartrate‐resistant acid phosphatase

## 1 Introduction

The equilibrium between osteoclast and osteoblast activities is crucial for maintaining healthy bone structure (Tuckermann and Adams 2021; Sánchez‐de‐Diego et al. 2021). When bone disorders such as osteoporosis, periodontitis, and ankylosing spondylitis cause an imbalance of osteoblastic and osteoclastic activity, they eventually damage the integrity of bone (Lee et al. 2021; Jacome‐Galarza et al. 2019). Postmenopausal elderly women suffering from osteoporosis, characterized by excessive osteoclast activity, can eventually develop life‐threatening fragility fractures (Zheng et al. 2022).

RANKL signaling is crucial for osteoclastic activity. In response to RANK–RANKL binding and macrophage colony‐stimulating factor (M‐CSF), osteoclast precursors originating from macrophages/monocytes fuse mutually to form large multinucleated osteoclast cells, causing bone resorption (Jacome‐Galarza et al. 2019). Mechanistically, RANKL binding to RANK triggers several signaling pathways, including NF‐κB and mitogen‐activated protein kinase (MAPK). Furthermore, NFATc1 is activated, promoting the expression of genes stimulating osteoclastogenesis. Research indicates that intracellular reactive oxygen species (ROS) are essential for osteoclast maturation and activation, in addition to RANKL signaling (Sun et al. 2020; Weng et al. 2021). Osteoporosis caused by estrogen deficiency is strongly linked to elevated ROS levels and increased osteoclast activity (Lee et al. 2021). Currently available anti‐osteoporosis medications bear significant limitations owing to their side effects. Targeting intracellular ROS could be a promising therapeutic approach for osteoporosis and related bone diseases.

Danshen, a long‐established traditional Chinese medicine preparation, has recently demonstrated beneficial effects on bone homeostasis (Guo et al. 2014). Salvianolic acid A (SAA), the chief active component in Danshen, has been found to exert various pharmacological effects, including antioxidant (Zhang et al. 2014, 2019), anti‐inflammatory (Lin et al. 2017; Wu, Wang, et al. 2020), and antithrombotic activities (Wu, Xu, et al. 2020). SAA is known to influence osteoblastogenesis, but its effects on osteoclastogenesis are not well understood. In the current study, we assessed whether SAA attenuates ROS levels to inhibit pathological osteoclastogenesis and prevent osteoclast‐related osteoporosis.

## 2 Materials and Methods

The SAA solution was purchased from MCE (Shanghai, China) and further diluted with culture medium to working concentrations. DMEM, α‐MEM, fetal bovine serum (FBS), and penicillin/streptomycin (P/S) solution were purchased from HyClone (Logan, UT, USA). Antibodies against NFATc1, cathepsin K (CTSK), and BTB and CNC homology 1 (Bach1) were purchased from Santa Cruz (Dallas, CA, USA). Antibodies against c‐Fos and mmp9, along with an NF‐κB Pathway Antibody Sampler Kit, were purchased from CST (Danvers, MA, USA). Antibodies against nuclear factor erythroid 2‐related factor 2 (Nrf2), HO‐1, p38, ERK, p‐ERK, and GAPDH were purchased from Proteintech (Wuhan, China). Antibodies against p‐JNK, JNK, and p‐p38 were purchased from Affinity Biosciences (Zhenjiang, China). Recombinant RANKL and M‐CSF were obtained from R&D Systems (Minneapolis, MN, USA). A Cell Counting Kit‐8 (CCK‐8) assay kit was sourced from MCE (Shanghai, China). The NE‐PER Nuclear and Cytoplasmic Extraction Reagents kit was sourced from Thermo Fisher (Rockford, IL, USA). The TRAcP Staining Kit and FAK Staining Kit were sourced from Sigma‐Aldrich (St. Louis, MO, USA). The ROS Assay Kit, Mitochondrial Superoxide Assay Kit, and the BCIP/NBT Alkaline Phosphatase Color Development Kit were sourced from Beyotime (Shanghai, China).

### 2.1 Cell Culture and BMMs Harvest

RAW264.7 cells (ATCC, Shanghai, China), a monocytic cell line derived from mice, were cultured in complete DMEM containing 10% FBS and 1% P/S. Primary BMMs were harvested from the tibiae and femurs of C57/BL6 mice aged 6–8 weeks. BMMs were cultured in α‐MEM with 50 ng/mL M‐CSF, 10% FBS, and 1% P/S. All cells were maintained under incubation conditions of 37°C with 5% CO_2_. The osteoclastogenic differentiation of RAW264.7 cells was assessed after a 3‐day period, whereas the differentiation of BMMs was evaluated after 5 days.

### 2.2 Cytotoxicity Assay and Molecular Docking

RAW264.7 cells (5 × 10^3^) and BMMs (1 × 10^4^) were cultured in 96‐well plates and exposed to specified concentrations of SAA for specific lengths of time. A 10 μL/well volume of CCK‐8 reagent was added, followed by a 1‐h incubation in the dark. The optical density (OD) was measured using spectrophotometric absorbance at 490 nm (BMG LABTECH, Ortenberg, Germany).

SAA (CID: 5281793) was fetched from PubChem and equipped by LigPrep; RANK (ID: 5BNQ Chain R) and RANKL (ID: 4GIQ Chain A) were obtained from the PDB database. The structures were prepared using the Protein Preparation Wizard, and the binding area was identified through Protein Interaction Analysis (PIPER pose energy: −288.629). During docking, the best‐scoring SAA on the RANK pose was identified using XP Gscore and MM‐GBSA.

### 2.3 In Vitro Osteoclastogenesis Assay (TRAcP) and Alkaline Phosphatase Staining

RAW264.7 cells and BMMs, properly seeded with 12‐h incubation, were induced by RANKL (100 ng/mL) and M‐CSF (50 ng/mL) alongside specified concentrations of SAA (10, 20, 30 μM). The culture medium was refreshed daily until mature osteoclasts developed in the control group. The cells were subsequently fixed and stained using the TRAcP Staining Kit. TRAcP‐positive multinucleated cells were identified as mature osteoclasts, and their quantity and size were quantified.

MC3T3‐E1 cells (1 × 10^3^) were cultured in an osteogenic medium with or without SAA (10, 30 μM). The activity of alkaline phosphatase (ALP) was assessed using the BCIP/NBT Alkaline Phosphatase Color Development Kit. The osteoblasts were prepared and stained with Alizarin Red S, and images were acquired using an inverted microscope.

### 2.4 FAK Staining

BMMs were properly seeded and cultured to differentiate into osteoclasts with and without SAA (10, 20, 30 μM). Osteoclasts fixed with paraformaldehyde were permeabilized using 0.1% Triton X‐100 for 5 min and subsequently blocked with 1% BSA for 30 min. Cells were cultured with anti‐vinculin (1:100) for 1 h, then treated with TRITC‐conjugated phalloidin in the dark for another 1 h. Finally, the nuclei were stained for 5 min with DAPI. Fluorescence microscopy (Leica, Germany) was used to visualize the cells.

### 2.5 Resorption Pit Assay

BMMs (1 × 10^4^) were cultured in 96‐well Osteo Assay Surface Plates (Corning) and differentiated into osteoclasts over 5 days, with or without specified SAA doses. For pit formation analysis, the cells were treated with sodium hypochlorite for 10 min and then washed with distilled water. Microscopic images of resorption pits were analyzed for absorption area using ImageJ software (Bethesda, USA).

### 2.6 Quantitative Real‐Time PCR

BMMs were induced by RANKL and M‐CSF with different doses of SAA (10, 20, and 30 μM) for 5 days. Isolated RNA was purified with TRIzol reagent (Thermo Fisher Scientific). cDNA was prepared using PrimeScript RT reagent Kit (Takara).RT‐qPCR was performed with SYBR Green PCR Master Mix in a CFX Connect (BIO‐RAD), with GAPDH serving as the internal control. The specific primers used are listed in Table S1. The expression of target genes was normalized to GAPDH's average expression (*n* = 5).

### 2.7 Detection of Intracellular ROS Production

Intracellular ROS and mitochondrial superoxide levels were quantitatively assessed utilizing the Reactive Oxygen Species Assay Kit and the MitoSOX Red reagent, respectively. In accordance with the kit instructions, BMMs were induced into mature osteoclasts without or with SAA (10 and 30 μM) for 5 days. Subsequently, the cells were incubated with DCFH‐DA for a duration of 20 min under dark conditions. Finally, the cells were imaged using a fluorescence microscope (Leica, Germany). For evaluation of early ROS generation in SAA‐pretreated BMMs, the cells were analyzed with a flow cytometer (BD Biosciences, USA) to record the ratio of fluorescence‐positive BMMs. The fluorescence intensity was quantified utilizing a microplate reader, with excitation and emission wavelengths set at 488 and 525 nm, respectively (BioTek, USA).

### 2.8 Measurement of Nuclear Translocation

To identify key cytokines involved in nuclear translocation in osteoclastogenesis, we performed immunofluorescence staining for NFATc1 and Nrf2. BMMs, in a good functional state, were pretreated with SAA (30 μM) for 3 h, followed by 3 h of stimulation. BMMs were treated with 4% paraformaldehyde for 30 min and subsequently blocked with a 4% BSA solution for 1 h. Anti‐Nrf2 and anti‐NFATc1 antibodies were incubated individually for 12–18 h at 4°C, and incubated with a secondary antibody conjugated to Alexa Fluor 633 or DyLight 488 in the dark for 2 h. Finally, fluorescence images with DAPI‐stained cell nuclei were acquired.

### 2.9 Preparation of Nuclear Protein Lysate

Nuclear and cytoplasmic proteins were extracted from BMMs using the NE‐PER kit as per the manufacturer's instructions. BMMs were lysed using cytoplasmic lysis buffer, and the supernatants containing cytoplasmic proteins were collected after centrifugation. The pellet was lysed using nuclear lysis buffer and centrifuged to obtain the nuclear protein supernatant.

### 2.10 Western Blot Assay

BMMs were collected and lysed in RIPA buffer for 30 min at 4°C to extract protein. Proteins were resolved using 8%–10% SDS‐PAGE (Beyotime Biotechnology, China) and subsequently transferred onto PVDF membranes (Bio‐Rad, USA). Following a 2‐h blocking with 5% skim milk, membranes were incubated with primary antibodies (1:1000) for 12 h at 4°C, then with HRP‐conjugated secondary antibodies for 2 h at room temperature. Protein bands were detected with enhanced chemiluminescence substrate (Sigma‐Aldrich, USA) and analyzed by ImageJ software (Bethesda, USA).

### 2.11 In Vivo Osteoporosis Mouse Model Induced by Ovariectomy

All experimental procedures were carried out adhering to the guidelines of the animal care and use committee of the Army Medical University (AMUWEC20213593). Eighteen C57BL/6J female mice (8 weeks old) were obtained from the Animal Experiment Center of the Army Medical University. A random sample of mice was divided into three groups: sham (*n* = 6), ovariectomy (OVX) (*n* = 6), and OVX + SAA (*n* = 6). In the sham group, bilateral ovaries were segregated and exposed, while bilateral OVX was carried out in the other two groups to induce osteoporosis. With a 1‐week recovery, the mice in the OVX + SAA group were administered SAA dissolved in normal saline (NS) (5 mg/kg/day) by gavage daily, while the mice in the other two groups were given equivalent amounts of NS for a total of 12 weeks. After humane euthanization of the mice, a midline abdominal incision was placed, through which the liver and kidney were harvested for morphological evaluation, and blood samples were collected from the heart for toxicity testing. Then, the left femurs were detached from the soft tissues for microstructural tests, while the right femurs were removed for morphological analysis.

### 2.12 Micro‐CT Scanning and Histomorphometric Femur Analysis

To analyze the microstructure, high‐resolution micro‐CT (Bruker microCT, Belgium) was employed to scan the left femurs. The trabecular bone (TB) region of interest (ROI) was manually delineated over a 3‐mm area starting 0.8 mm proximal to the femoral epiphysis. Analyses in both 3D and 2D were conducted using CT Analyser software (Version 1.15.4.0, Belgium). CTAn software was used to analyze the bone parameters. After decalcification in 10% EDTA for over 3 weeks, the right femurs were embedded in paraffin and sliced into 4‐μm sections. The sections were stained with hematoxylin and eosin (H&E) and Masson stain to visualize bone tissue, while TRAcP staining was performed to evaluate osteoclasts. Liver and kidney sections were assessed by H&E staining.

### 2.13 Statistical Analysis

All representative data are presented as the mean ± standard deviation (SD). All in vitro experiments were independently repeated more than three times. Student's *t*‐test and one‐ or two‐way analysis of variance (ANOVA) with Bonferroni's correction were used to evaluate the differences between two groups and for multiple comparisons, respectively. Statistical significance was defined as a *p* < 0.05.

## 3 Results

### 3.1 SAA Retards RANKL‐Mediated Osteoclastogenesis

**FIGURE 1:**
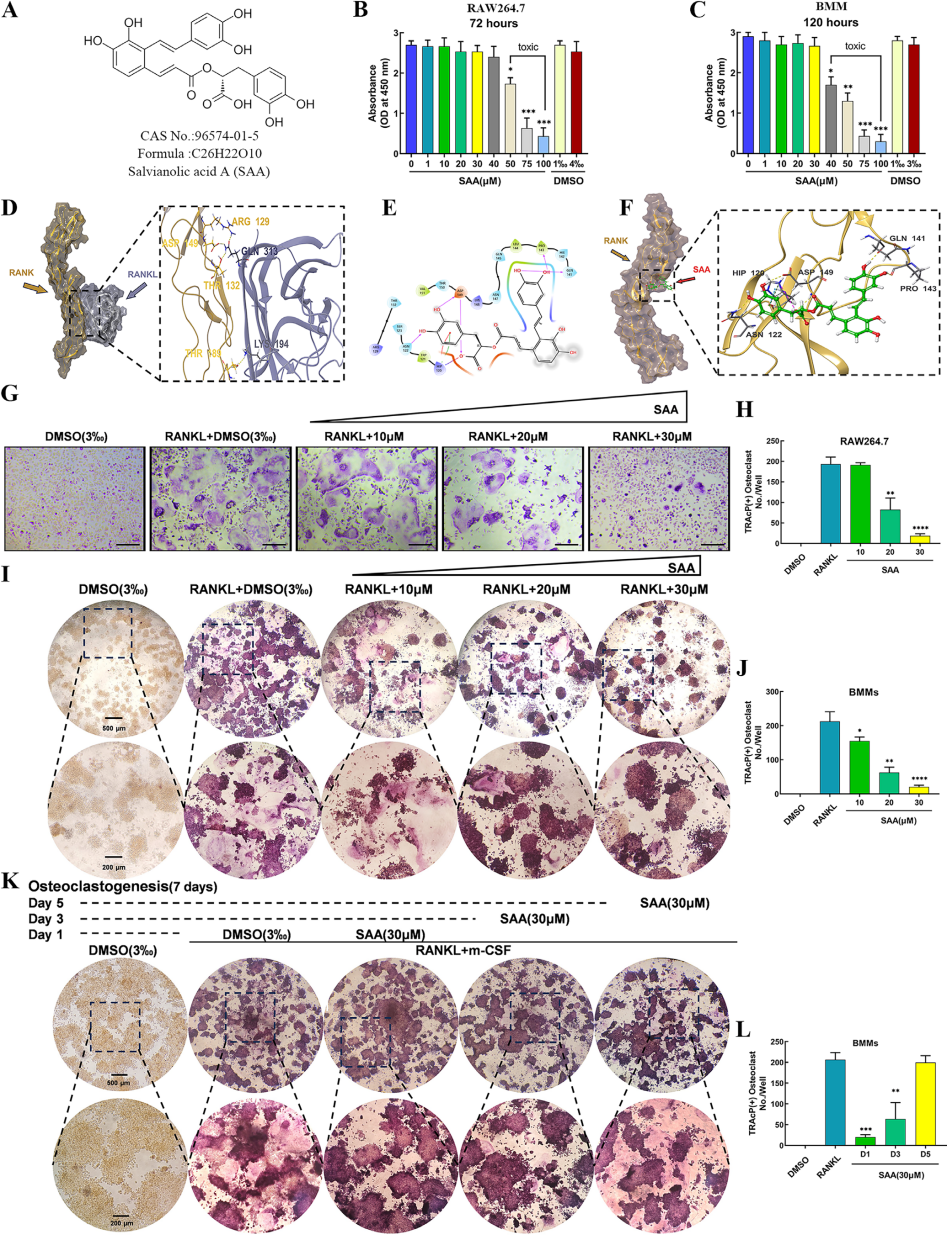
SAA suppresses osteoclastogenesis induced by RANKL and M‐CSF in vitro. (A) SAA's chemical structure and formula are presented. (B and C) Viability of RAW264.7 cells or BMMs tested by CCK‐8 assay after treatment with the indicated dose of SAA for 72 or 120 h. (D) A 3D RANK–RANKL complex model was developed to explore SAA's interaction with RANK. (E) Images showing the potential SAA binding site on the surface of the active pocket of RANK. (F) Structural 3D image showing the strong affinity bonding of SAA and RANK (yellow: hydrogen bonds; red: salt bridge, blue: π–π bond, and green: π–cation). (G) Images of TRAcP staining demonstrating that SAA dose‐dependently inhibited 3‐day osteoclastogenesis of RAW264.7 cells. (H) Quantification of mature multinucleated (nuclei > 3) osteoclasts (*n* = 3). (I and J) Concentration‐dependent suppression by SAA of BMM osteoclastogenesis induced by 5 days of treatment with RANKL and M‐CSF. (K) TRAcP staining images depict BMMs treated with 30 μM SAA during osteoclastogenesis. (L) Quantification of TRAcP‐positive multinucleated osteoclasts after treatment with SAA (*n* = 3). Data are presented as the mean ± SD. *p < 0.05, **p < 0.01, ***p < 0.001, ****p < 0.0001. Scale bar = 200 μm.

The chemical structure of SAA is presented in Figure 1A. To assess the toxicity of SAA, BMMs and RAW264.7 cells were treated with the specified concentrations of SAA for a specific length of time and examined by CCK‐8 assays. During osteoclastogenesis, SAA showed no cytotoxicity to BMMs and RAW264.7 cells up to 30 μM (Figures 1B,C and S1A,B). To elucidate the relationship between SAA and RANK, a computational molecular docking analysis was conducted, which demonstrated a strong interaction between SAA and RANK (Figure 1D–F). The strong affinity (XP Gscore: −6.065; MM‐GBSA dG Bind: −57.4 kcal/mol) may have hindered the interaction between RANK and RANKL. To assess SAA's impact on osteoclastogenesis, RAW264.7 cells and BMMs were cultured in 96‐well plates with varying SAA concentrations. SAA inhibited the differentiation of RAW264.7 cells into TRAcP‐positive multinucleated osteoclasts in a dose‐dependent manner, as observed in the control group (Figure 1G,H). Furthermore, SAA inhibited RANKL and M‐CSF‐induced osteoclastogenesis in BMMs (Figure 1I,J). BMMs were treated with 30 μM SAA at the indicated time points to determine the stage that was affected (Figure 1K). A primary suppressive effect of SAA was seen during the early stages (Days 1–3) of osteoclastogenesis, in contrast to the mid‐late stage (Days 3–6) (Figure 1L).

### 3.2 SAA Impairs Podosome Belt Formation and Osteoclastic Resorption In Vitro

**FIGURE 2:**
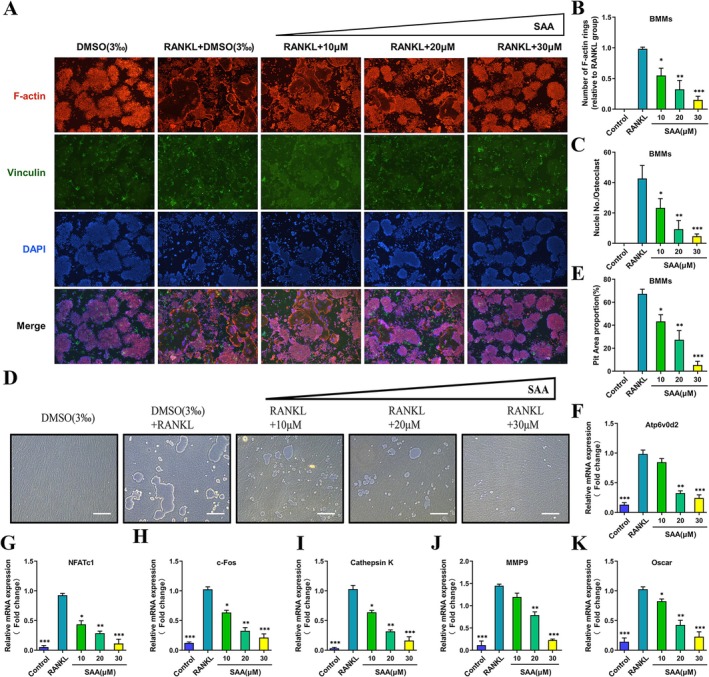
SAA hinders osteoclast fusion and resorption and suppresses osteoclast marker gene expression. (A) Representative immunofluorescence images of FAK staining showing podosome belt formation in BMMs induced by RANKL and M‐CSF with various doses of SAA for 5 days. F‐Actin was stained with tetramethylrhodamine‐conjugated phalloidin (red); focal contacts were stained with vinculin mAb (green); nuclei were stained with DAPI (blue). (B and C) Quantification of the relative areas of osteoclasts and the number of nuclei per osteoclast (*n* = 5). (D) Representative images of osteoclast resorption by BMMs cultured in hydroxyapatite‐coated plates for 5 days with or without SAA. (E) Quantification of the resorbed pit area and hydroxyapatite area per well (*n* = 5). (F–K) RT‐qPCR analysis of the expression of osteoclast‐specific genes, including Atp6v0d2 (F), Nfatc1 (G), c‐Fos (H), cathepsin K (I), MMP9 (J), and Oscar (K) relative to GAPDH (*n* = 5). Data are presented as the mean ± SD. *p < 0.05, **p < 0.01, ***p < 0.001, ****p < 0.0001. Scale bar = 200 μm.

As podosome belt formation is the characteristic feature of functional mature osteoclasts, we treated BMMs with varying doses of SAA and then performed FAK staining to visualize the morphological changes. In the control group, typical intact podosome belt structures with multitudinous nuclei were formed, while SAA, by comparison, significantly minimized the size and number of podosome belt structures proportionally with dose (Figure 2A–C). Next, we tested whether SAA impaired osteoclast resorptive activity with a resorption pit assay. An evident reduction in the resorption area was observed in the SAA‐treated group (Figure 2D). In addition, a quantitative analysis of resorption pit ratios in the Osteo Assay plate indicated that SAA inhibited resorption of osteoclasts in a dose‐dependent manner (Figure 2E). During osteoclastogenesis, several osteoclast‐specific genes, such as c‐Fos, Nfatc1, Ctsk, Atp6v0d2, Oscar, and Mmp9, are upregulated. Using quantitative PCR, we assessed the mRNA levels of these genes to evaluate the effect of SAA and discovered that SAA inhibited all of them in a dose‐dependent manner (Figure 2F–K). Collectively, these available data further confirmed that SAA could restrain the formation of multinucleated OCs and impair osteoclastic resorption in vitro.

### 3.3 SAA Mitigates NFATc1 Activity and Its Downstream Factors

**FIGURE 3:**
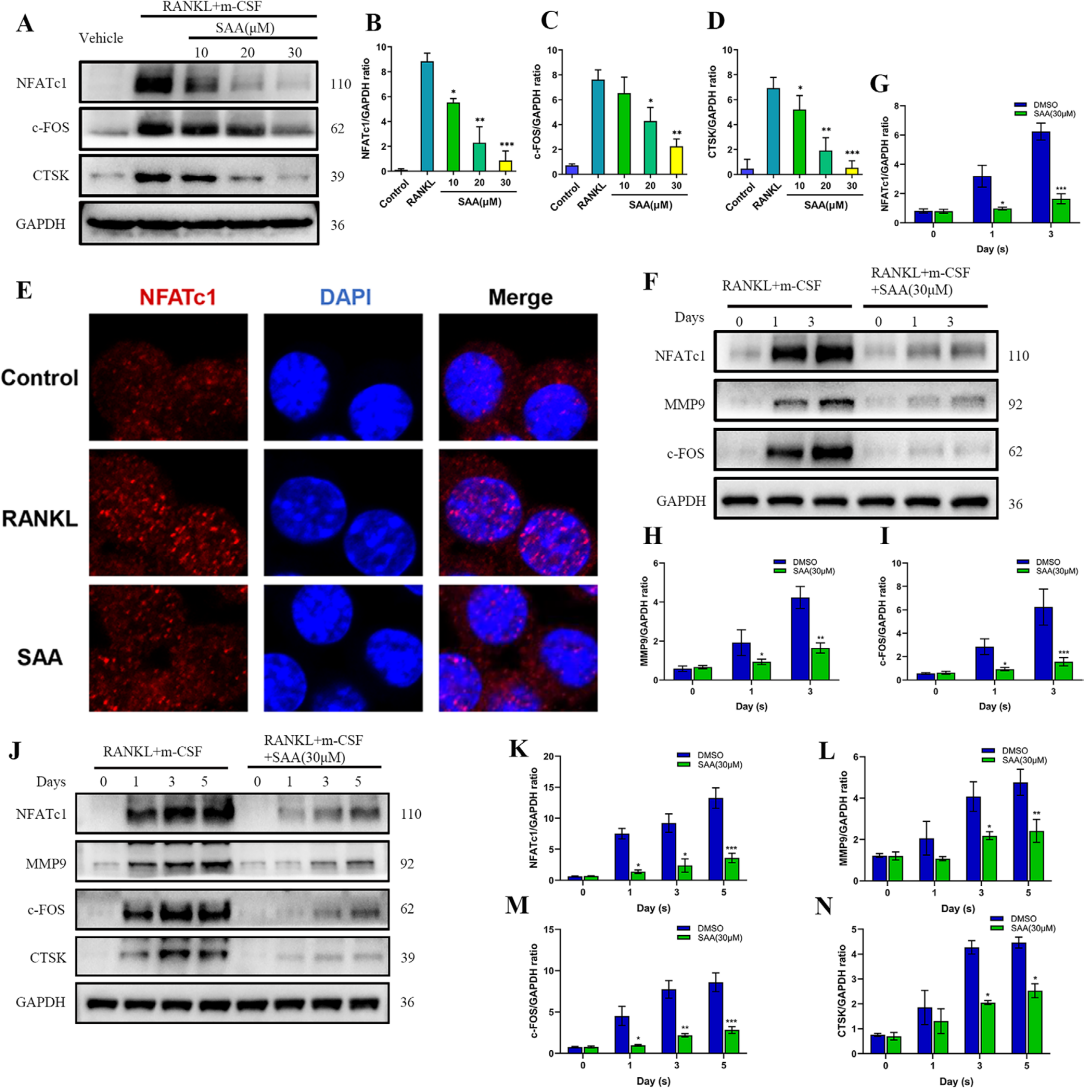
SAA hinders the activation of NFATc1 signaling during osteoclastogenesis. (A–D) BMMs were induced with various doses of SAA for 5 days. The expression levels of key osteoclast‐specific proteins were tested by Western blotting. (E) Representative confocal microscopy immunofluorescence images showing that SAA (30 μM) restricts the nuclear translocation of NFATc1 during osteoclastogenesis. Scale bar = 20 μm. (F–N) The protein levels of NFATc1 and other osteoclast‐distinct proteins during osteoclastogenesis. RAW264.7 cells (F) and BMMs (J) were induced with or without SAA (30 μM) for the indicated times, and protein was collected for Western blotting. (G–I) Quantification of the protein levels of NFATc1, c‐Fos, and MMP9 in RAW264.7 cells normalized to GAPDH (*n* = 3). (K–N) Quantification of the protein levels of NFATc1, c‐Fos, MMP9, and CTSK in BMMs normalized to GAPDH (*n* = 3). All data are presented as the mean ± SD. *p < 0.05, **p < 0.01, ***p < 0.001.

NFATc1 serves as the principal transcriptional regulator, playing an indispensable role during osteoclastogenesis (Shin et al. 2015). Previously, we observed that the transcription of NFATc1 and its downstream marker genes was abrogated significantly in a dose‐related manner by SAA. We evaluated SAA's effect on NFATc1 and downstream protein expression through Western blot (WB) analysis (Figure 3A). Consistent with previous PCR results, SAA dose‐dependently restricted the activity of NFATc1 (Figure 3B) and its downstream marker factors related to osteoclast formation and function, such as c‐Fos (Figure 3C) and CTSK (Figure 3D). Nuclear translocation of NFATc1 is crucial for osteoclastogenesis. We assessed SAA's effect on NFATc1 nuclear translocation using immunofluorescence staining and detected overt inhibition (Figure 3E). In addition, during the whole period of osteoclastogenesis, the protein level of NFATc1 and its downstream osteoclast‐specific factors was downregulated by SAA treatment, including in RAW264.7 cells (Figure 3F–I) and BMMs (Figure 3J–N). Consequently, these data confirmed that SAA could strongly inhibit osteoclastogenesis by lessening NFATc1 activity, together with downstream protein expression.

### 3.4 SAA Disrupts RANKL‐Mediated Actuation of the NF‐κB and MAPK Pathways

**FIGURE 4:**
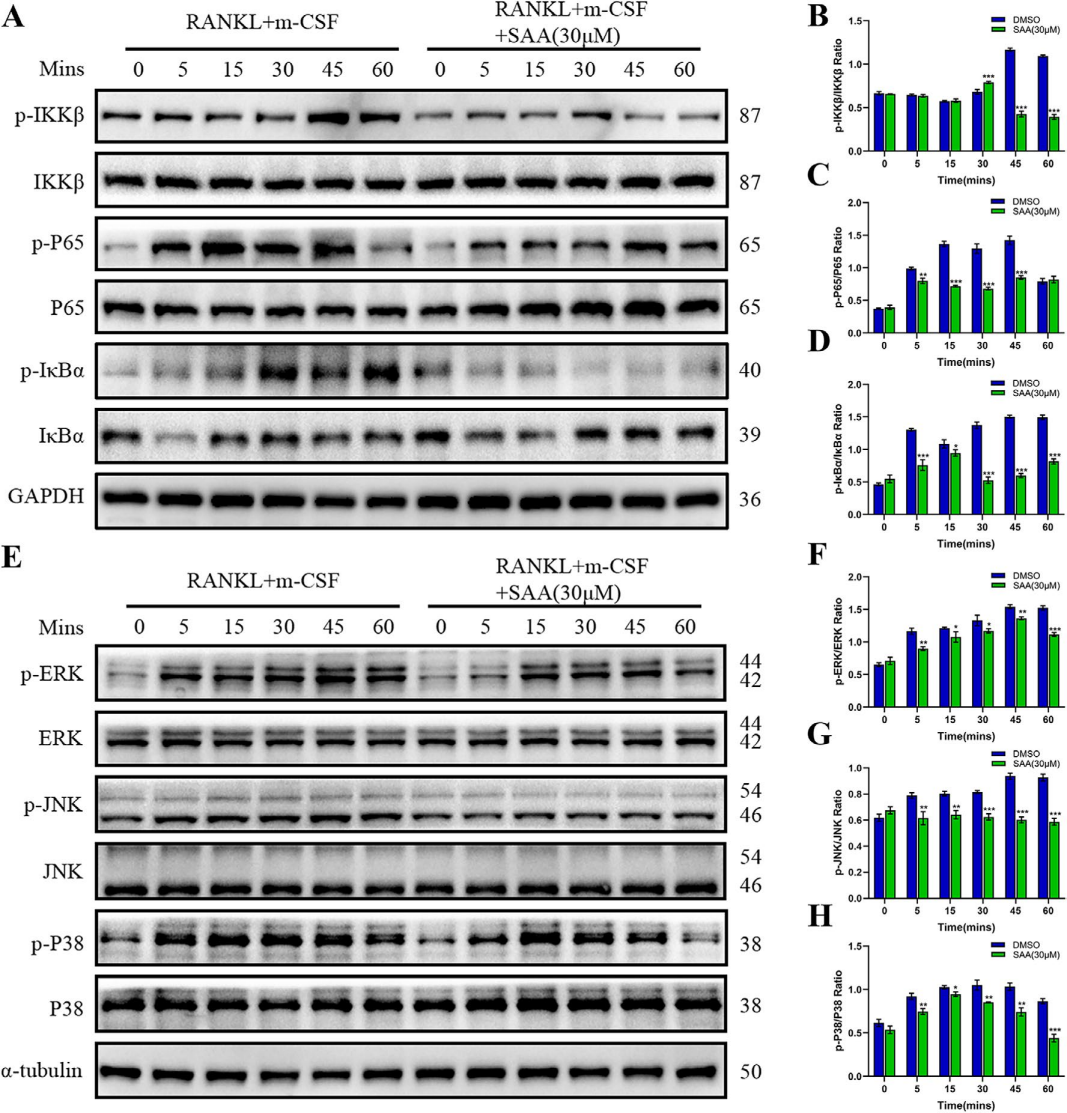
SAA retards osteoclast differentiation by curbing the NF‐κB and MAPK pathways activation. (A) Representative image of the expression of the proteins involved in NF‐κB signaling in BMMs induced with or without SAA (30 μM). (B–D) Quantification of the proportion of phosphorylated/unphosphorylated IκB, p65, and IKKβ relative to GAPDH (*n* = 3). (E) Representative Western blotting images of the impact of SAA on the proteins involved in the MAPK pathway, including ERK, p38, and JNK. (F–H) Quantification of the proportion of phosphorylated/unphosphorylated proteins normalized to GAPDH (*n* = 3). All data are presented as the mean ± SD. *p < 0.05, **p < 0.01, ***p < 0.001.

During osteoclastogenesis induced by RANKL and M‐CSF, the key underlying mechanisms are the classical NF‐κB and MAPK pathways (Park‐Min et al. 2014). Upon RANKL stimulation, IκB‐α is phosphorylated and degraded to start signaling. However, SAA lessened the activation of IκB‐α and NF‐κB P65 (Figure 4A,C,D). Moreover, the key regulatory step, phosphorylation of IκB kinase‐β (IKK‐β), was diminished by SAA (Figure 4B). Then, we tested the impact of SAA on the MAPK signaling pathway (Figure 4E). Compared to the control, the respective phosphorylation ratio of total MAPK markers (ERK, p38, and JNK) decreased significantly after SAA treatment (Figure 4F–H). In summary, these data demonstrated that SAA distinctly suppressed the NF‐κB and MAPK pathways triggered by RANKL and M‐CSF.

### 3.5 SAA Lowers RANKL‐Induced ROS by Activating Nrf2/HO‐1 and Boosting Antioxidant Enzymes

**FIGURE 5:**
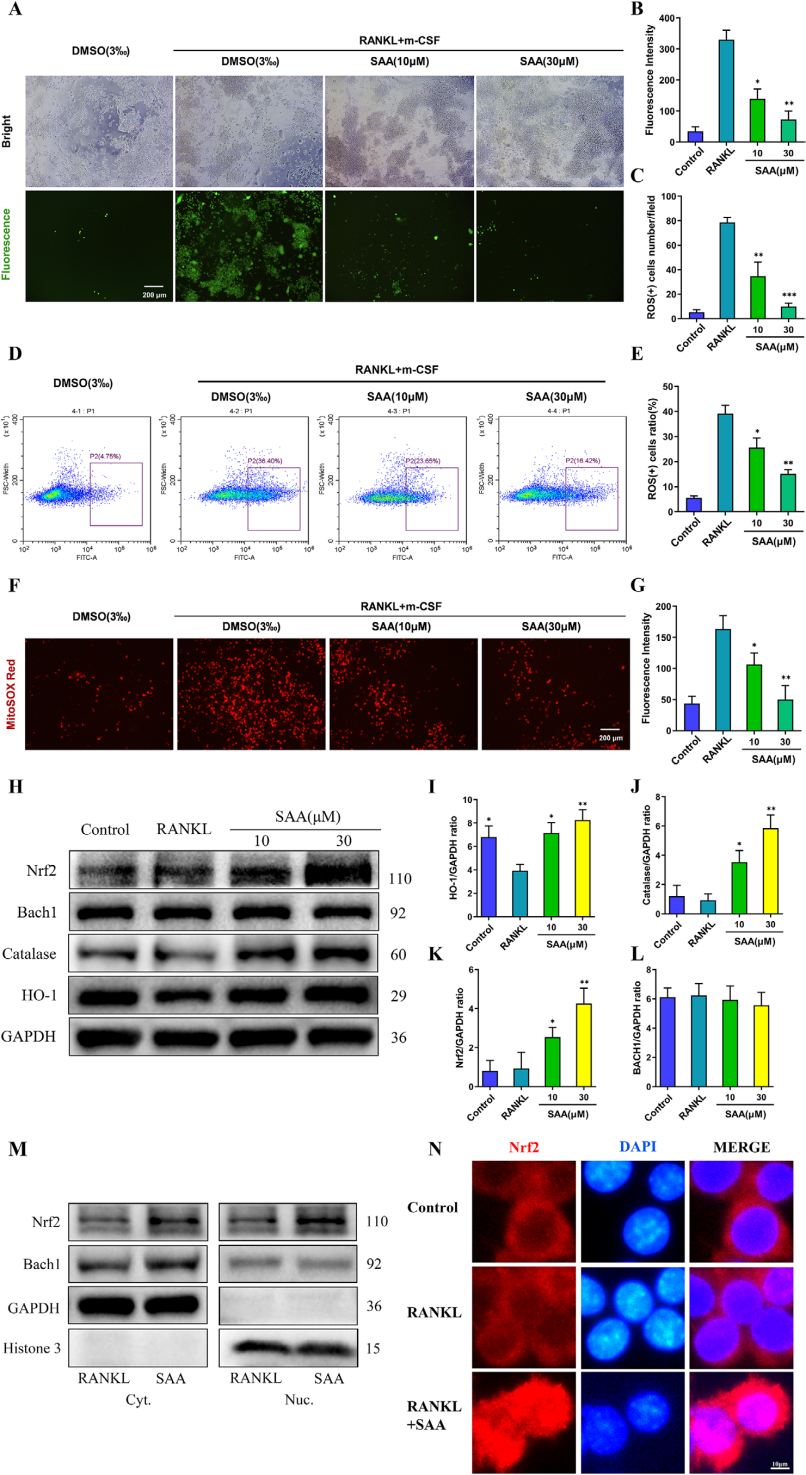
SAA attenuates ROS generation and enhances Nrf2/HO‐1 signaling. (A) Fluorescence images showing intracellular ROS generation in BMMs with or without 5‐day SAA pretreatment at different doses. Scale bar = 200 μm. (B) Quantification of the average fluorescence intensity of DCF (*n* = 3). (C) Quantification of the average number of ROS‐positive cells (*n* = 3). (D and E) Flow cytometry analysis of the instant impact of SAA on ROS generation. (F) Fluorescence images showing mitochondrial superoxide production in BMMs, pretreated or not with different SAA doses. Scale bar = 200 μm. (G) Quantification of the average fluorescence intensity of MitoSOX Red (*n* = 3). (H) Representative images of the impact of SAA on the protein expression of key antioxidant enzymes, including Nrf2, Bach1, HO‐1, and catalase. (I–L) Analysis of the ratios of Nrf2, Bach1, HO‐1, and catalase to GAPDH (*n* = 3). (M) The expression of Nrf2 and Bach1 in the cytosol (Cyt.) and nuclear (Nuc.) fractions was detected by Western blotting after treatment with SAA. GAPDH (Cyt.) and histone H3 (Nuc.) were used as loading controls. (N) Fluorescence microscopy images showing SAA facilitated Nrf2's nuclear translocation. Scale bar = 20 μm. All data are presented as the mean ± SD. *p < 0.05, **p < 0.01, ***p < 0.001.

Increasing evidence suggests that intracellular ROS levels are crucial for osteoclast differentiation (Kim et al. 2017). We investigated how SAA influences ROS production during osteoclastogenesis. Intracellular ROS were detected using the cell‐permeable, oxidation‐sensitive dye H2DCFDA (Pucino et al. 2019). BMMs induced by RANKL and M‐CSF presented a very large number of ROS‐positive cells, which was visibly decreased by SAA (Figure 5A). A dose‐related reduction in DCF fluorescence was observed in BMMs in the presence of SAA (Figure 5B,C). To pinpoint when SAA inhibited ROS production during osteoclastogenesis, flow cytometry was used to assess the ROS positivity ratio in BMMs at the start of induction (Figure 5D,E). Our results indicated that SAA inhibited osteoclastogenesis by suppressing the generation of intracellular ROS. To determine if mitochondria were responsible for ROS, MitoSOX Red reagent was used. RANKL stimulation of osteoclasts pretreated with SAA at 10 and 30 μM showed reduced MitoSOX Red fluorescence, suggesting reduced levels of mitochondrial superoxide (Figure 5F,G).

To unravel the underlying mechanisms by which SAA affects ROS generation, we investigated the expression of antioxidant enzymes through WB analysis (Kanzaki et al. 2016). The expression of HO‐1 was diminished by RANKL stimulation, but this effect was reversed and even enhanced by SAA treatment (Figure 5H,I). Similarly, SAA also improved the expression of catalase (Figure 5H,J). HO‐1 expression and enzyme activity mostly depend on transcriptional regulation of Nrf2 and Bach1 (Sanada et al. 2021). The protein level of Nrf2 was notably enhanced by SAA treatment, without a significant change in the expression of Bach1 (Figure 5H,K,L). Furthermore, we found that SAA could promote Nrf2 protein levels in the cytoplasm and its translocation to the nucleus with less of an impact on Bach1 (Figure 5M). In addition, representative immunofluorescence staining demonstrated the intensive nuclear translocation of Nrf2 (Figure 5N). Based on these data, it could be inferred that SAA suppresses intracellular ROS levels during osteoclastogenesis through the Nrf2/HO‐1 pathway to promote antioxidant enzyme expression.

### 3.6 SAA Diminishes Bone Loss in an OVX‐Induced Osteoporotic Mouse Model

**FIGURE 6:**
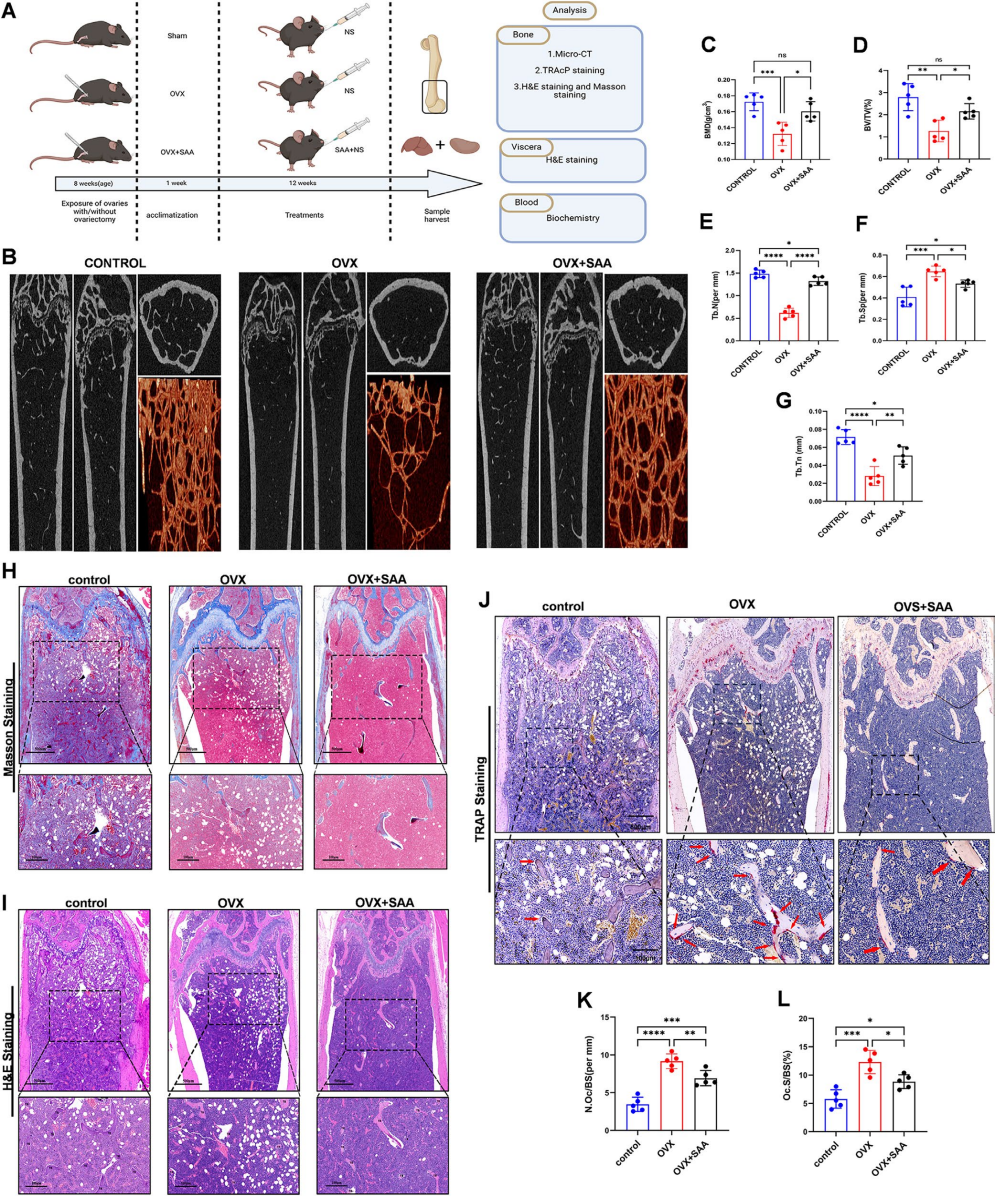
SAA treatment prevents bone loss in the osteoporotic mouse model. (A) Schematic depiction of the ovariectomy‐induced osteoporotic mouse model setup and experimental framework. Created with BioRender.com. (B) Typical micro‐CT image showing the prevention of bone loss by SAA administration. (C–G) Bone microstructure parameters, including BMD, BV/TV, Tb.N, Tb.Sp, and Tb.Th, were quantified (*N* = 5 per group). (H–J) Representative images of Masson, H&E, and TRAP staining of decalcified bone slices. (K and L) Quantification of N.Oc/BS and Oc.S/BS (*n* = 5). All data are presented as the mean ± SD. *p < 0.05, **p < 0.01, ***p < 0.001, ****p < 0.0001. Scale bar = 500 μm.

Given SAA's ability to inhibit osteoclast formation and function in vitro, we investigated its therapeutic potential in an OVX‐induced mouse model of osteoporosis. Micro‐CT revealed that the structure of the trabeculae of distal femurs was severely damaged in ovariectomized (OVX) mice compared to sham‐operated mice, and SAA prevented bone deterioration in osteoporotic mice (Figure 6A). Moreover, quantitative analysis showed that SAA treatment significantly increased bone mineral density (BMD) and improved bone parameters in OVX mice, including BV/TV, trabecular number (Tb.N), trabecular thickness (Tb.Th), and trabecular separation (Tb.Sp) values (Figure 6B–G). Consistently, histological assessment and Masson staining showed that with SAA treatment, the severity of bone destruction induced by OVX was greatly improved (Figure 6H,I). To confirm whether SAA prevents bone loss via osteoclasts in vivo, we performed TRAcP staining on isolated femur bone slices (Figure 6J). Quantification of the osteoclast parameters demonstrated that SAA could remarkably diminish the number and surface area of TRAcP‐positive cells around the trabecula relative to OVX mice (Figure 6K,L). These results confirmed that SAA rescued estrogen deficiency‐induced bone loss by suppressing the activity of osteoclasts. To test the toxicity of SAA, we performed blood tests and observed no serious adverse events (Figure S2A). Histological analysis of the vital metabolic organs revealed no pathological microstructural changes in the liver or kidney (Figure S2B). All of the in vivo data demonstrated that SAA could prevent bone loss in estrogen‐deficient mice without hepatorenal toxicity.

## 4 Discussion

Osteoporosis, characterized by brittle and fragile bones, mainly occurs in elderly postmenopausal women (Reid 2021). Consequently, antiresorptive therapy, targeting osteoclasts, predominates among the therapies for osteoporosis. An increasing number of effective medications have been applied in clinical practice, such as estrogen replacement treatment, bisphosphonates, denosumab, teriparatide, and strontium ranelate (Reid and Billington 2022). However, even at a safe dose, these medications may result in severe adverse effects such as hepatoxicity, nephrotoxicity, cancer, and osteonecrosis (Compston et al. 2019). A safe and efficacious alternative drug is thus urgently needed. SAA exerts bone protective effects by promoting osteogenesis and repressing adipogenesis against prednisone (GC)‐induced osteoporosis (Cui et al. 2009). And SAA accelerated the healing of delayed fractures in vivo in the bone‐targeting liposome (BTL) formulation (Liu et al. 2018). SAA has been validated to have an osteoprotective effect in osteoblasts (Liu et al. 2018) and chondrocytes (Zhou et al. 2020). Consistent with the above study, we found the same results but with a reduced effect of a relatively large dose of SAA (Figure S3A–F). For the first time, we demonstrated that SAA significantly retarded the development of osteoclasts in vitro and halted bone loss in an OVX‐induced osteoporosis mouse model. Therefore, SAA influences bone balance by enhancing osteogenesis and inhibiting osteoclastogenesis.

RANKL binding to RANK on osteoclast precursor cells is a vital step that initiates osteoclastogenesis and guarantees a population of functional osteoclasts (Ikebuchi et al. 2018). In vitro, RAW264.7 cells and BMMs were successfully induced to differentiate into mature functioning osteoclasts in response to RANKL and M‐CSF. SAA greatly restrained in vitro osteoclastogenesis in a dose‐ and time‐dependent manner. Osteoclast‐specific genes and proteins, detected by RT‐PCR and WB, further verified the negative regulation of osteoclastogenesis by SAA. However, SAA had no significant effect on mature osteoclasts (Figure S4A–D).

To explore the fundamental mechanisms underpinning this negative regulation, we evaluated the key signaling pathways involved. Accumulating evidence indicates that NF‐κB signaling is critical for initiating osteoclastogenesis (Jimi et al. 2004). Mice with defective NF‐κB signaling present an osteoporotic phenotype with impaired osteoclast differentiation (Iotsova et al. 1997). The NF‐κB pathway is activated by phosphorylating IκBα at Ser32 and Ser36, causing its ubiquitination, degradation, and allowing NF‐κB p65 to move to the nucleus. We found that SAA effectively suppressed IκB phosphorylation and decreased NF‐κB p65 nuclear translocation, which was verified by immunoblot analysis and immunofluorescence staining. The IKK complex determines IκB phosphorylation, and mice lacking the IKKβ gene exhibit irreversible osteogenesis disorders (Ruocco et al. 2005). We assessed SAA's effect on IKKβ activation and found that SAA slowed osteoclastogenesis by inhibiting IKK phosphorylation.

Apart from canonical RANKL signaling, another vital mechanism involved in osteoclastogenesis is the MAPK pathway. RANKL and M‐CSF stimulation phosphorylates ERKs, enhancing the transcription of key osteoclastogenic factors c‐Fos and NFATc1 (Lee et al. 2018). Meanwhile, the other key family members, p38 and JNKs, are activated to promote the expression of factors like TRAP and CTSK, which are linked to osteoclast differentiation and resorption. Considering that SAA substantially retarded the expression of osteoclast‐specific genes and proteins, we evaluated SAA's effect on the MAPK pathway. Similarly, SAA notably repressed the activation of the MAPK pathway.

NFATc1, as a pivotal transcriptional regulator, orchestrates the expression of genes like c‐Fos and CTSK, crucial for osteoclast differentiation. The deficiency of NFATc1 causes impaired osteoclastogenesis and osteopetrosis in vivo (Song et al. 2009). Our study showed that SAA could inhibit osteoclastogenesis by reducing NFATc1 expression and nuclear translocation. During osteoclastogenesis, NF‐κB signaling and NFATc1 can be regulated by intracellular ROS as upstream molecules to promote the transcription of osteoclast‐instinct genes (Kim et al. 2018). Accumulating studies have suggested that endogenous ROS, well‐regulated by Nrf2/Keap1/ARE signaling axis, are closely linked to osteoclastogenesis (Chen et al. 2020). Once the Nrf2/Keap1/ARE signaling axis is activated, Nrf2 breaks up with Keap1 and enters the nucleus to boost the expression of antioxidant enzymes to eliminate ROS (Qi et al. 2023). During osteoclastogenesis, RANKL induces Bach1 nuclear import to attenuate Nrf2 activity by occupying ARE sequences, and then attenuating expression of antioxidant enzymes like NQO1 and HO‐1, thereby augmenting intracellular ROS signaling (Kanzaki et al. 2014; Wada et al. 2020). With SAA treatment, the expression of several ROS scavengers, such as HO‐1 and catalase, was upregulated. Transcriptional regulation of the HO‐1 gene mainly depends on the interaction between Nrf2 and Bach1 (Sanada et al. 2021). In this study, we found that SAA promoted the expression of Nrf2 and its nuclear translocation. In contrast, SAA showed little impact on the inhibitory transcription factor Bach1. Our findings indicate that SAA impedes RANKL‐induced ROS production and inhibits the nuclear translocation of NFATc1 by activating the Nrf2/HO‐1 antioxidant pathway.

Given the strong negative effects of SAA on osteoclastogenesis observed in vitro, we investigated its impact on osteoporosis in an OVX mouse model. It is widely accepted that OVX mice can simulate osteoporosis seen in the elderly postmenopausal population (Li et al. 2020). Notably, SAA exhibited a prominent bone protective effect in vivo. Micro‐CT and histological analyses revealed that SAA treatment preserved TB structure and reduced bone volume loss in OVX mice. A lower number of TRAP‐positive osteoclasts was found in the bone of SAA‐treated mice, which was consistent with the data noted in vitro. These findings suggest that SAA could be a promising new treatment for osteoporosis and osteoclast‐related disorders. Moreover, in SAA‐treated mice, there was no evident liver or kidney damage observed.

## 5 Conclusion

**FIGURE 7:**
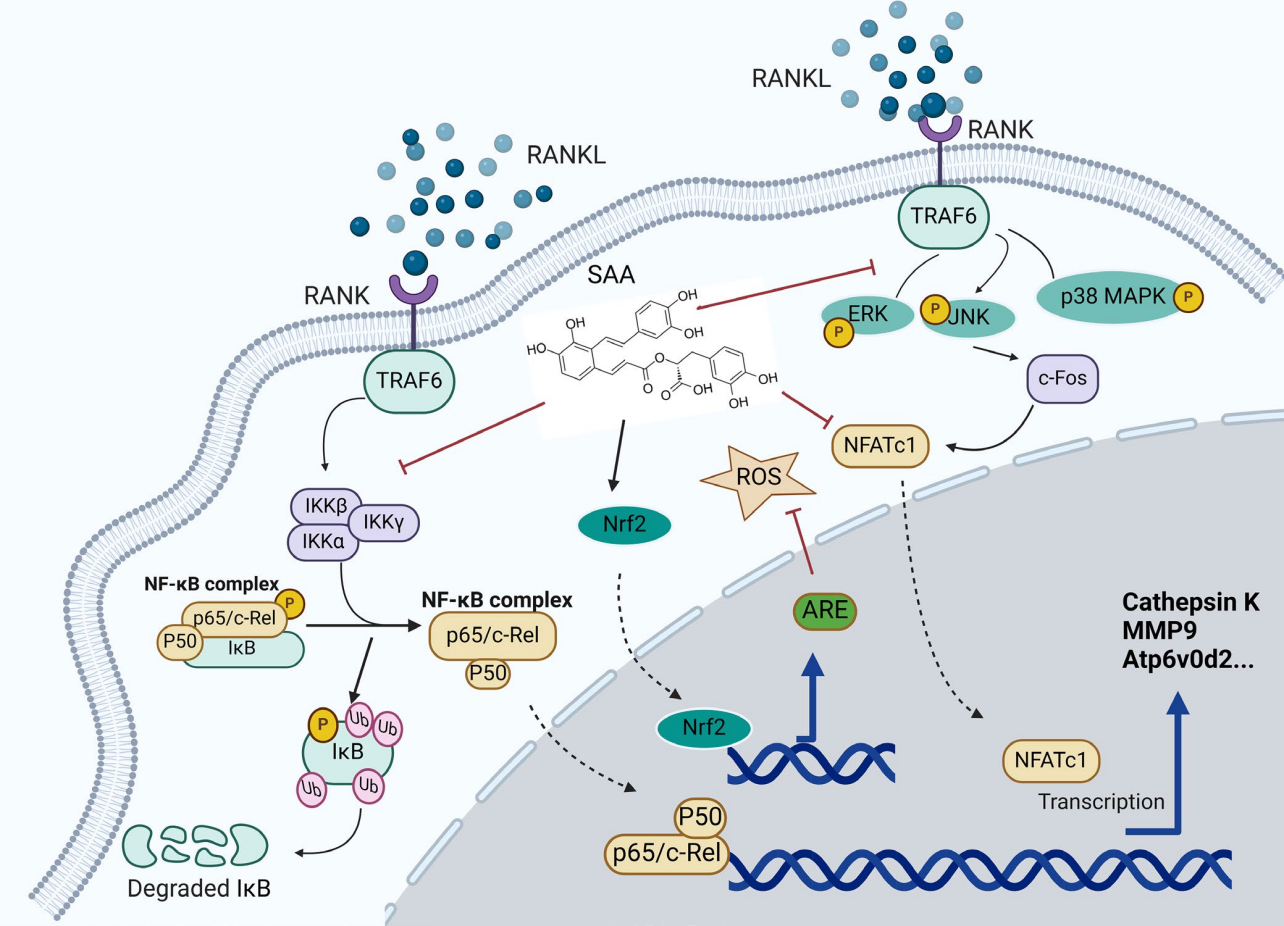
An overview of the molecular regulation by SAA during osteoclastogenesis induced by RANKL. RANKL binding to RANK activates NF‐κB and MAPK signaling, leading to NFATc1 nuclear translocation and amplification, which initiates osteoclastogenesis. However, all three key pathways could be effectively blocked by SAA treatment. In addition, SAA attenuated RANKL‐induced ROS accumulation by boosting Nrf2 expression and its movement to the nucleus. Adapted from “NF‐κB Signaling Pathway,” by BioRender.com (2022). Retrieved from https://app.biorender.com/biorender‐templates.

Considering all available data, we conclude that SAA significantly suppresses RANKL‐mediated osteoclastogenesis and mitigates bone loss in osteoporotic mice without toxicity. Furthermore, SAA stifles not only the NF‐κB, MAPK and NFATc1 pathways, but also restricts ROS generation via the Nrf2/HO‐1 axis during the early stages of osteoclastogenesis. An illustration of possible mechanisms by which SAA suppresses osteoclastogenesis is presented in Figure 7. Our findings suggest that SAA may be an effective treatment for osteoclast‐mediated osteoporosis.

## Author Contributions

**Hao Qiu:** conceptualization, data curation, formal analysis, methodology, visualization, writing – original draft. **Chenhui Cai:** data curation, methodology, visualization. **Ying Zhang:** formal analysis, investigation. **Sizhen Yang:** methodology, validation. **Xu Hu:** data curation, validation. **Tongwei Chu:** conceptualization, funding acquisition, supervision, writing – review and editing.

## Ethics Statement

All animal‐related experimental protocols were approved by the Laboratory Animal Welfare and Ethics Committee of the Third Military Medical University (protocol number: AMUWEC20213593).

## Consent

We have obtained consent to publish this paper from all the participants of this research.

## Conflicts of Interest

The authors declare no conflicts of interest.

## Supporting information


**Data S1.** Supporting Information.

## Data Availability

All data generated or analyzed during this study are included in this published article.
