# Supplementary material for: Salvianolic Acid A Mitigates Osteoporotic Bone Loss by Repressing Reactive Oxygen Species via the Nrf2–HO‐1 Pathway
Source: Phytother Res. 2025 Aug 6;39(11):4977–90. doi: 10.1002/ptr.8503 (PMC12605832; doi:10.1002/ptr.8503)
Supplement: Supplementary file 1 — Data S1. Supporting Information. [file PTR-39-4977-s001.docx]

**Supplementary Table 1.** Primer sequences for qRT-PCR.

| Genes | Forward | Reverse |
| --- | --- | --- |
| GAPDH | 5’-GGTTGTCTCCTGCGACTTCA-3’ | 5’-TGGTCCAGGGTTTCTTACTCC-3’ |
| CTSK | 5’-GGGAGAAAAACCTGAAGC-3’ | 5’-ATTCTGGGGACTCAGAGC-3’ |
| c-Fos | 5’-GATGAGAAGTCTGCGTTGC-3’ | 5’-CTCTGGGAAGCCAAGGT-3’ |
| MMP9 | 5′-CGTGTCTGGAGATTCGACTTGA-3′ | 5′-TTGGAAACTCACACGCCAGA-3′ |
| NFATc1 | 5’-CCTTCAGAGAGACCTTGGC-3’ | 5’-CACAGGAGCTGGGGTTC-3’ |
| OSCAR | 5′-CTGCTGGTAACGGATCAGCTCCCCAGA-3′ | 5′-CCAAGGAGCCAGAACCTTCGAAACT-3′ |
| ATP6V0d2 | 5′-GTGAGACCTTGGAAGACCTGAA-3′ | 5′-GAGAAATGTGCTCAGGGGCT-3′ |


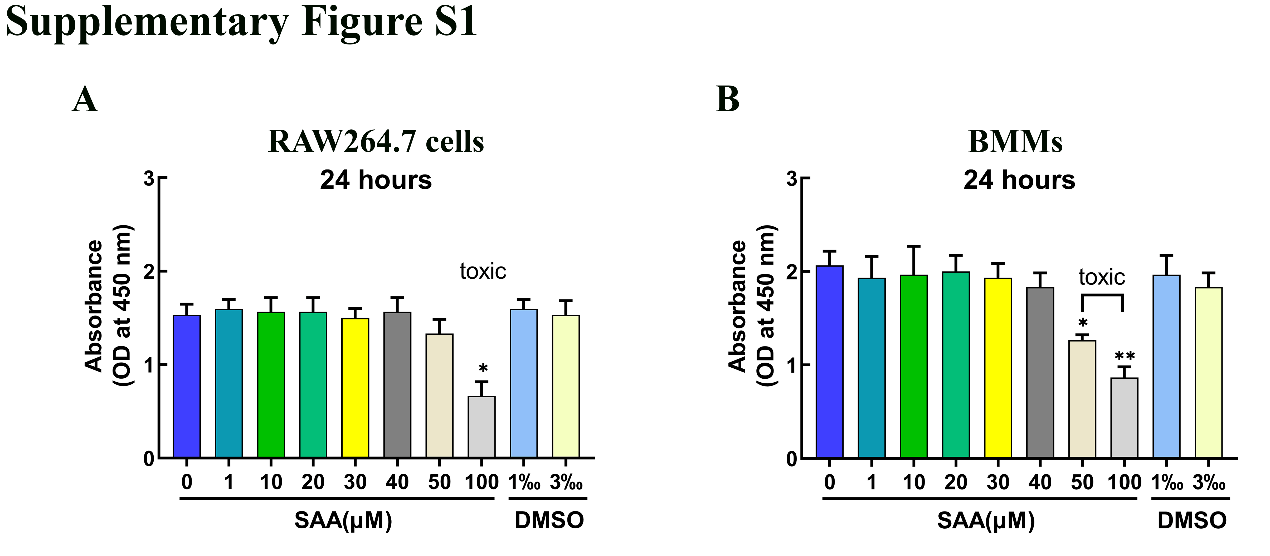


Supplementary figure S1. (A and B) RAW264.7 cells and BMMs were treated with specific SAA concentrations for 24 hours, and cell viability was measured using the CCK8 assay. ALL data are presented as the mean±SD(n=5). *P<0.05, **P<0.01.


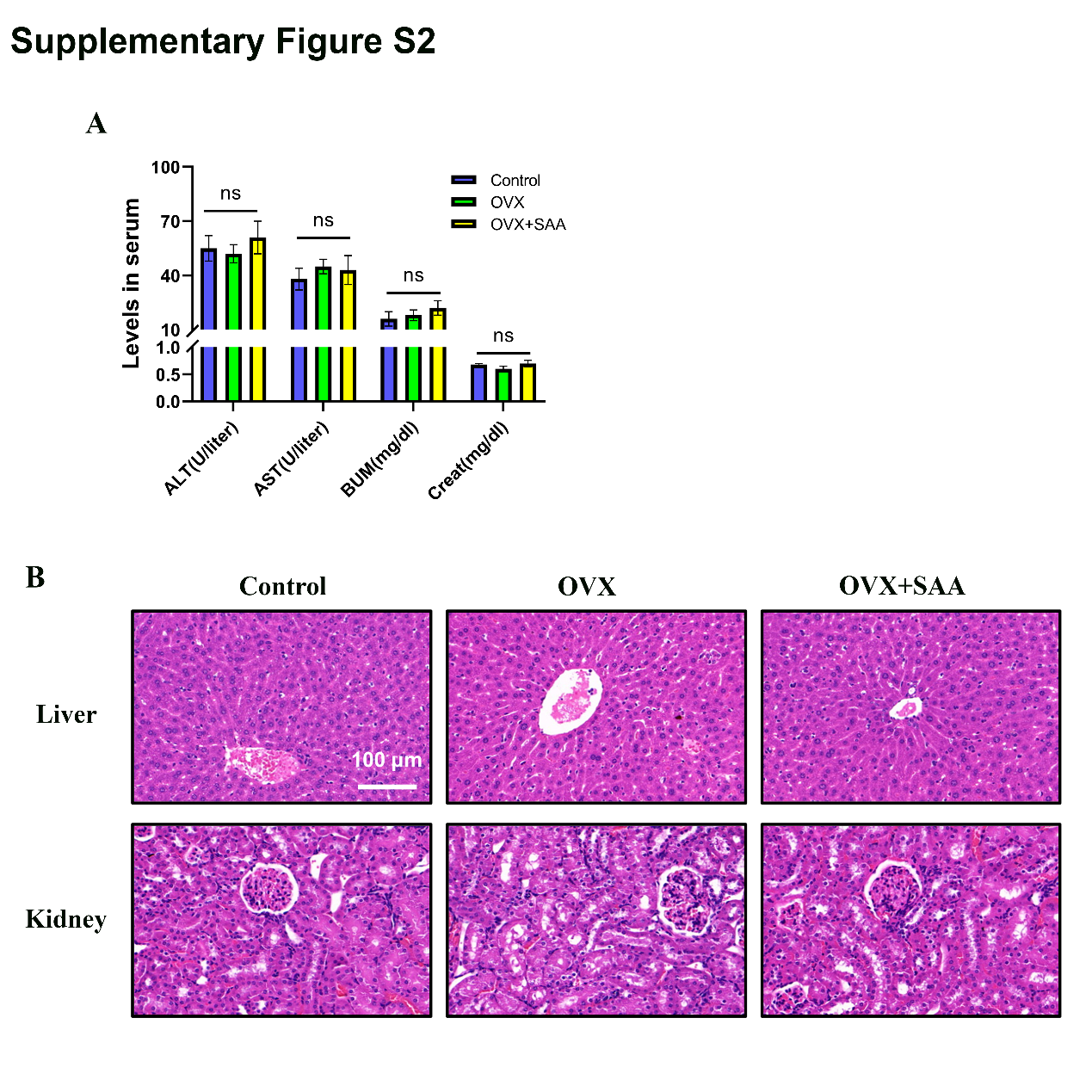


Supplementary figure S2. (A) Representative photomicrographs of the liver and kidney histological sections from OVX mice; (B) Serum biochemical profiles of the mice. ALT, alanine ami-notransferase; AST, aspartate aminotransferase; Creat, creatinine; BUN, blood urea nitrogen. All data are presented as the mean± SD(n=3). ns for no significance, *P<0.05, **P<0.01, ***P<0.001.


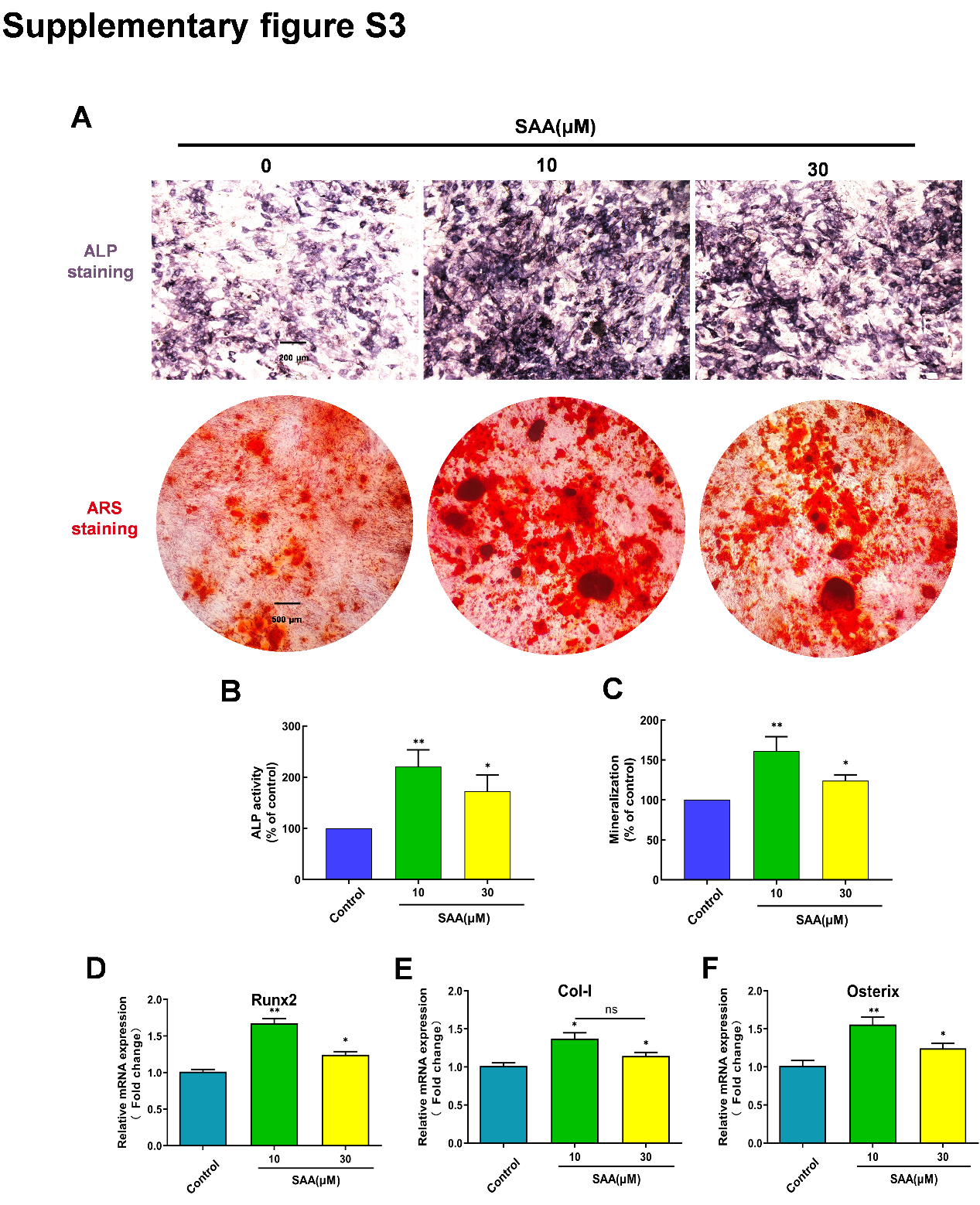


Supplementary figure S3. (A, B) Representative photomicrographs depict ALP activity and mineralization in MC3T3-E1 cells following a 2-week osteogenic induction with different SAA concentrations, alongside their quantification results. (D-F) qRT-PCR analysis was conducted to assess the expression levels of osteoblast-specific genes: Runx2, Col-1, and Osterix. Gene expression was normalized to GAPDH. All data are presented as the mean± SD(n=5). ns for no significance, *P<0.05, **P<0.01, ***P<0.001.


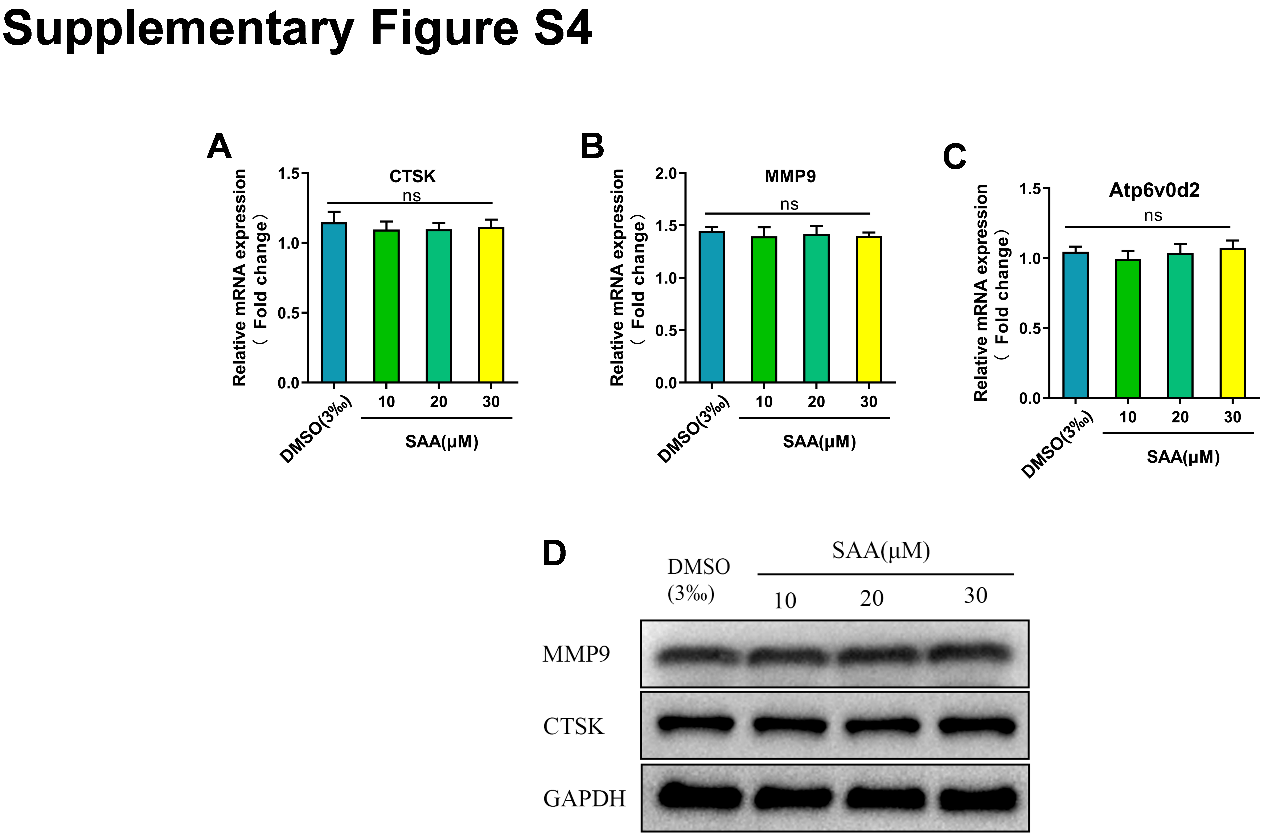


Supplementary figure S4. (A-C) qRT-PCR analysis evaluated the expression of osteoclast-specific genes CTSK, CMMP9, and Atp6v0d2, relative to GAPDH, in mature osteoclasts exposed to different concentrations of SAA. (D) representative Western blot images of CTSK and MMP9 proteins in mature osteoclasts induced from BMMs with various dose of SAA treatment. All data are presented as the mean± SD(n=5). ns for no significance, *P<0.05, **P<0.01, ***P<0.001.
